# Effects of synbiotics containing *Bifidobacterium animalis* subsp. *lactis* GCL2505 and inulin on intestinal bifidobacteria: A randomized, placebo‐controlled, crossover study

**DOI:** 10.1002/fsn3.1033

**Published:** 2019-04-21

**Authors:** Daisuke Anzawa, Takashi Mawatari, Yoshiyuki Tanaka, Mio Yamamoto, Tomomi Genda, Shota Takahashi, Tomohiko Nishijima, Hiroshi Kamasaka, Satoru Suzuki, Takashi Kuriki

**Affiliations:** ^1^ Institute of Health Sciences Ezaki Glico Co., Ltd. Osaka Japan; ^2^ Shinagawa Season Terrace Health Care Clinic Tokyo Japan

**Keywords:** *Bifidobacterium animalis* subsp. *lactis*, intestinal bifidobacteria, randomized trial, synbiotics

## Abstract

A number of studies have shown the bifidogenic effects of either probiotic bifidobacteria or inulin, and this bifidogenic shift in the composition of the colonic microbiota is likely the basis for their positive impact on human health. This study aimed to evaluate the effects of synbiotics containing the probiotic bacterium *Bifidobacterium animalis* subsp. *lactis* (*B. lactis*) GCL2505 and inulin on the levels of intestinal bifidobacteria compared with *B. lactis* GCL2505 alone. A randomized, double‐blind, placebo‐controlled, crossover trial was carried out involving 60 healthy subjects with a tendency for constipation using fermented milk containing *B. lactis* GCL2505 and inulin (synbiotic), only *B. lactis* GCL2505 (probiotic), and placebo. Fecal samples were collected at the end of each 2‐week intervention period, and the bifidobacterial count was analyzed by quantitative real‐time PCR. The numbers of total bifidobacteria and *B. lactis* in feces were significantly increased during the probiotic and synbiotic intake periods compared with the placebo intake period. Furthermore, the numbers of total bifidobacteria and endogenous bifidobacteria were significantly higher in the synbiotic intake period compared with the probiotic intake period, while there was no difference in the number of *B. lactis*. These results suggested that the synbiotics containing *B. lactis* GCL2505 and inulin had a greater effect on the number of bifidobacteria than a drink containing probiotics alone and could be useful for the improvement of the intestinal environment.

## 1 INTRODUCTION

Several hundred different types and over 100 trillion bacteria inhabit the human intestinal tract, and they form a complex community of microbes (Finegold, Sutter, & Matheisen, 1983). In recent years, many studies have indicated that there is a relationship between microbiota and various diseases of the host human, with an individual's microbiota playing a role in diseases such as obesity, diabetes, inflammation, and autism spectrum disorder (Blander, Longman, Iliev, Sonnenberg, & Artis, 2017; Larsen et al., 2010; Ley, Turnbaugh, Klein, & Gordon, 2006; van De Sande, van Buul, & Brouns, 2014). It has also been reported that changes in the microbiota, including a reduction of diversity and shifts in the composition ratio of intestinal bacteria, play a key role in host health (Ley et al., 2005; Manichanh et al., 2006; Rajilic‐Stojanovic et al., 2011).

Members of the genus *Bifidobacterium* are one of the most predominant organisms in the human intestinal tract and are important for general health, which means that their diversity and abundance provide markers for measuring the stability of the human intestinal microbiota, as well as the overall intestinal environment (Mitsuoka, 1984; Tanaka, 1995). Since bifidobacteria, regardless of the species, have various positive features for host health, including the production of vitamins, polyphenols, conjugated linoleic acids, and lactate/acetate as well as the enhancement of gut barrier function and the immune system (Rivière, Selak, Lantin, Leroy, & De Vuyst, 2016), many attempts have been made to increase the number of intestinal bifidobacteria. An increase in the number of bifidobacteria is thought to improve the intestinal microflora and environment, which not only prevents the deterioration of fecal characteristics, constipation, and diarrhea, but also benefits systemic health, such as metabolic disease, atopic disease, or irritable bowel syndrome (Kalliomäki et al., 2001; Kerckhoffs et al., 2009; Malinen et al., 2005; Schwiertz et al., 2010). Therefore, it can be said that the improvement of the intestinal environment by maintaining a high number of bifidobacteria in the intestine is important for the maintenance and promotion of good health, and numerous attempts to increase the number of intestinal bifidobacteria in the human intestinal tract have been made.

A definition of probiotics has been proposed as “live microorganisms that, when administered in adequate amounts, confer a health benefit on the host” (Hill et al., 2014). Many probiotic strains, mainly bifidobacteria and lactic acid bacteria, have been studied and demonstrated to exert various health benefits. Especially, some probiotic strains increase the number of intestinal bifidobacteria, which contribute to the improvement of intestinal disorders such as constipation (Matsumoto et al., 2010; Yaeshima et al., 1997; Yamano et al., 2006). To induce a greater effect on the number of intestinal bifidobacteria, another approach has been proposed that combines probiotics with prebiotics. Prebiotics are nonviable food components that confer a health benefit on the host associated with modulation of the gut microbiota (Pineiro et al., 2008), and are expected to be utilized by endogenous bifidobacteria and to increase their number in the intestinal tract. The combination of both probiotics and prebiotics is called synbiotics (Gibson & Roberfroid, 1995). The ingestion of synbiotics is also expected to lead to a large increase in the number of bifidobacteria in the intestinal tract (Childs et al., 2014; Krumbeck et al., 2018; Macfarlane, Cleary, Bahrami, Reynolds, & Macfarlane, 2013; Shioiri et al., 2006). Unfortunately, because previous reports evaluated the bifidogenic effects of synbiotics compared with only placebo, not with probiotics directly, and studies did not result in a significant increase in the number of bifidobacteria compared with probiotics, it has not been shown definitively that synbiotics are clearly better than probiotics or prebiotics at increasing the number of bifidobacteria in the gut.

*Bifidobacterium animalis* subsp. *lactis* (*B. lactis*) GCL2505 is a probiotic strain derived from healthy human intestines. We previously revealed that *B. lactis* GCL2505 has some positive effects on health, such as an improvement of defecation frequency and a reduction of visceral fat (Aoki et al., 2016; Ishizuka et al., 2012; Takahashi et al., 2016; Tanaka et al., 2015). It is thought that these effects are attributable to a unique feature of *B. lactis* GCL2505, which can reach the intestine in a viable form and is able to proliferate after a single ingestion. This leads to an increase in the number of intestinal bifidobacteria. Combining *B. lactis* GCL2505 with prebiotics was expected to be more effective in increasing intestinal bifidobacteria, which would then lead to an enhancement of health benefits such as an improvement of defecation frequency. The purpose of the present study was to evaluate the effects of synbiotics using *B. lactis* GCL2505 on changes in intestinal bifidobacteria counts and defecation frequency, compared with those of *B. lactis* GCL2505 alone. In this study, we used inulin as a prebiotic, which is a fructan‐type, soluble dietary fiber (Mensink, Frijlink, van der Voort Maarschalk, & Hinrichs, 2015), and has a bifidogenic effect by which it is assimilated selectively into bifidobacteria in the intestine (Bouhnik et al., 2007; Rao, 2001). We designed a placebo‐controlled randomized, double‐blind, three‐group crossover study in healthy adults with a tendency for constipation to investigate the changes in the counts of *B. lactis* and nine different endogenous bifidobacteria in feces by quantitative real‐time PCR with *Bifidobacterium* species‐ and subspecies‐specific primers as the primary outcome and the frequency of defecation as the secondary outcome.

## 2 MATERIALS AND METHODS

### 2.1 Test food

The test products were fermented milk containing inulin (Orafti GR; BENEO GmbH, Mannheim, Germany) and *B. lactis* GCL2505 (synbiotic drink), only *B. lactis* GCL2505 (probiotic drink), or placebo. The inulin content of the synbiotic drink was 2.0 g/100 g, and the viable cell count of *B. lactis* GCL2505 in the synbiotic drink and probiotic drink was 1 × 10^10^ colony‐forming units (cfu)/100 g. The placebo was prepared with the same ingredients and adjusted for flavor and pH by adding food‐grade acetic acid and lactic acid similarly to the other test products. Their basic ingredients were skim milk powder, high‐fructose corn syrup, apple juice, starch syrup, yeast extract, flavor, acidulant, stabilizer, and sweetener. The nutritional details of the test drinks are shown in Table 1.

**Table 1:** Nutritional details of the test drinks

	Placebo	Probiotic drink	Synbiotic drink
Functional substance	—	*B.* * lactis* GCL2505^a^	*B. lactis* GCL2505^a^ and inulin^b^
Nutritional components (values for 100 g test drink)			
Energy (kcal)	42	42	45
Protein (g)	3.1	3.1	3.1
Fat (g)	0	0	0
Carbohydrate (g)	7.3	7.3	9.2
NaCl (g)	0.14	0.14	0.14
Ca (mg)	104	104	104

a 1 × 10^10^ cfu/100 g test drink.

b 2.0 g/100 g test drink.

### 2.2 Study design

The study was designed as a randomized, double‐blind, placebo‐controlled, three‐group crossover intervention trial. It was conducted according to the Consolidated Standards of Reporting Trials statement (Schulz, Altman, & Moher, 2011), the principles of the Declaration of Helsinki, and the Ethical Guidelines for Medical and Health Research Involving Human Subjects issued by the Ministry of Health, Labour and Welfare of Japan. The study protocol was approved by the institutional review board of the Ethics Committee of Nihonbashi Cardiology Clinic (Tokyo, Japan). The subjects provided written informed consent before initiation of the study. This study was performed by a contract research organization, KSO Corporation (Tokyo, Japan), from February to April 2018 at the Shinagawa Season Terrace Health Care Clinic (Tokyo, Japan) and registered as UMIN000031101 in the University Hospital Medical Information Network (UMIN) Clinical Trials Registry.

### 2.3 Subjects

Healthy Japanese participants aged 20–64 years with a tendency for constipation (3–5 days a week) were recruited. The exclusion criteria were as follows: (a) regular use of intestinal drugs and laxatives; (b) regular intake of healthy food to relieve constipation; (c) intake of food containing viable bacteria, such as lactic acid bacteria, bifidobacteria, and natto bacteria, and/or enhanced with oligosaccharides and dietary fiber, and/or healthy food to relieve constipation (including Food for Specified Health Uses [FOSHU]), and/or containing a large amount of sugar alcohol; (d) use of medicine that affects digestion and absorption such as antibiotics; (e) food allergy; (f) pregnancy, lactation, or planning to become pregnant; (g) serious diseases requiring urgent treatment or with severe complications; (h) a medical history of diseases or surgeries affecting digestion, absorption, and bowel movements; (i) a current or history of drug dependence and/or alcoholism; (j) participation in other clinical trials; (k) judgment of unsuitability for this study based on the subject's responses to a lifestyle questionnaire; and (l) judgment of unsuitability for this study by the principal investigator on the basis of clinical laboratory test results. Eligible subjects kept a daily record of defecation, ingestion of healthy food, usage of medicine, and physical condition before the screening test, in which the subjects received anthropometric measurements, clinical laboratory tests, and a medical interview. After analyzing the daily record and screening test, a total of 60 subjects were selected. An independent clinician randomly allocated the subjects into three groups (20 subjects/group) stratified for age, sex, and days of defecation according to the information obtained until the screening test. The groups were defined as group A: intake of the test drinks in the order of placebo, probiotic drink, and synbiotic drink; group B: intake of the test drinks in the order of probiotic drink, synbiotic drink, and placebo; and group C: intake of the test drinks in the order of synbiotic drink, placebo, and probiotic drink. The link between identification number and treatment group was kept in a sealed document by the allocation officer. The investigators, subjects, and study statistician were blinded to the allocation of the treatment groups until after all data analyses were completed.

### 2.4 Study protocol

**Figure 1:**
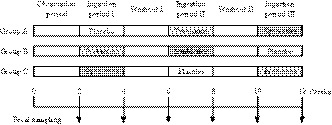
Study design

This study consisted of six periods of 2 weeks each, and the periods were as follows: observation period, ingestion period I, washout I, ingestion period II, washout II, and ingestion period III (Figure 1). During the ingestion periods, each subject consumed 100 g of the indicated test drink every day without setting a time for ingestion. The test drinks were delivered every week and stored in a refrigerator by the subjects. Throughout the study, the subjects were instructed to: (a) keep a daily record of test drink consumption, defecation, fecal status, ingestion of healthy food, usage of medicine, and physical condition; (b) maintain their regular lifestyle such as food and exercise (to avoid undereating, overeating, and overexercising); (c) avoid the overconsumption of alcohol; (d) use as few medicines as possible that affect digestion and absorption such as antibiotics; and (e) avoid the intake of food containing viable bacteria, such as lactic acid bacteria, bifidobacteria, and natto bacteria, and/or enhanced with oligosaccharides and dietary fiber, and/or healthy food to relieve constipation (including FOSHU), and/or containing a large amount of sugar alcohol. Clinical surveys were conducted at the start of each ingestion period and at the end of the study, in which the subjects received anthropometric measurements, clinical laboratory tests, and a medical interview. Feces were sampled at the end of each period and delivered to the laboratory in a refrigerated, anaerobic state using an AnaeroPack Kenki (Mitsubishi Gas Chemical Co., Inc., Tokyo, Japan). These samples were diluted 10‐fold with phosphate‐buffered saline (pH 7.4) and homogenized. Suspensions were kept at −80°C until required for analysis.

### 2.5 Gut bifidobacteria

Bacterial DNA was extracted from 10‐fold dilutions of the fecal samples, and the number of gut bifidobacteria was subsequently determined by quantitative real‐time PCR using *Bifidobacteria* species‐ and subspecies‐specific primers according to a procedure described previously (Tanaka et al., 2015). Total counts of bifidobacteria in the fecal samples are represented as the sum of 10 species (*B. longum* subsp. *longum*, *B. adolescentis*, *B. catenulatum*, *B. pseudocatenulatum*, *B. breve*, *B. bifidum*, *B. longum* subsp. *infantis*, *B. dentium*, *B. angulatum*, and *B. lactis*). Endogenous bifidobacteria were regarded as the sum of nine species, without *B. lactis*. The detection limit of each species or subspecies was 2.0 × 10^5^ cells per gram of feces.

### 2.6 Statistical analysis

From our previous studies, we assumed that the mean of differences in total bifidobacteria counts after intake of each test drink was 0.3 logarithm with a standard deviation of 0.5 logarithm. It was calculated that a sample size of 59 subjects would provide 80% power to detect a significant difference in means assuming that the common standard deviation was 0.5 logarithm using a *t* test with a 0.05 two‐sided significance level after the Bonferroni correction. On the basis of this calculation, we selected a total of 60 subjects in this crossover study.

The number of each *Bifidobacterium* species was expressed and analyzed after common logarithmic transformation. All measured values are expressed as the mean ± standard deviation. For the validation of the crossover, we carried out analysis of variance with the number of total bifidobacteria in the feces and evaluated the timing effect and order effect. Between‐group comparisons were conducted by Student's paired *t* test. Statistical significance was determined as *p* < 0.05 after the Bonferroni correction. All statistical analyses were performed using IBM SPSS Statistics for Windows software version 24 (IBM Corp., Armonk, NY, USA).

## 3 RESULTS

### 3.1 Subjects

**Figure 2:**
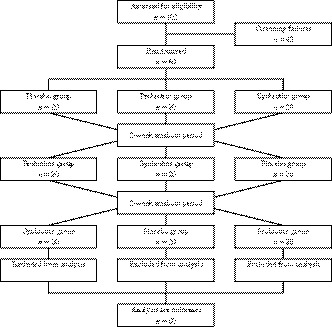
Flow diagram of the study showing numbers of participants

The study outline is shown in Figure 2. From among the 103 participants who provided written informed consent and completed the screening test, we selected 60 subjects who had relatively low defecation frequencies (3–5 days a week) and who did not meet the exclusion criteria. The background characteristics of the subjects are shown in Table 2. The subjects were allocated randomly to three groups, among which there was no significant difference in any baseline data (data not shown). All subjects completed the study and were incorporated into the efficacy analysis. No adverse events were observed in any subject throughout this study.

**Table 2:** Baseline characteristics of the subjects

	Total
N	60
Male/female	17/43
Age (years)	45.0 ± 8.9
Height (cm)	162.1 ± 6.5
Body weight (kg)	59.2 ± 9.5
Body mass index (kg/m^2^)	22.5 ± 3.0
Systolic blood pressure (mmHg)	119.9 ± 13.7
Diastolic blood pressure (mmHg)	75.0 ± 9.5
Pulse rate (beats/min)	76.4 ± 11.5
*Bifidobacterium* (log cells/g feces)	9.72 ± 0.89
Number of defecations (/week)	3.72 ± 0.63
Days of defecation (/week)	3.57 ± 0.58

Note

Values are expressed as the mean ± standard deviation.

### 3.2 Gut bifidobacteria

The time effect and order effect of each ingestion period for the number of total bifidobacteria in the intestinal tract were not statistically significant (*p* = 0.597 and *p* = 0.403, respectively). Thus, we concluded that the carryover effect could be ignored and the results obtained from the crossover design of the present study could be evaluated appropriately.

The number of gut bifidobacteria is shown in Table 3. In the probiotic intake period, there was a significant increase in the total count of bifidobacteria compared with the placebo intake period. Regarding the individual bifidobacteria species, the counts of *B. lactis*in the probiotic and synbiotic intake periods were significantly increased compared to the placebo intake period, while the number of endogenous bifidobacteria and *B. longum* was significantly decreased. In the synbiotic intake period, the amount of total bifidobacteria was increased significantly compared to the placebo and probiotic intake periods, and the number of *B. longum*, *B. adolescentis*, and endogenous bifidobacteria was increased compared with the probiotic intake period.

**Table 3:** Number of intestinal bifidobacteria

	Total bifidobacteria	lac	lon	ado	cat	bre	bif	inf	den	ang	Endogenous bifidobacteria
Placebo	9.78 ± 0.79	5.69 ± 0.87	9.12 ± 1.22	7.92 ± 2.09	7.95 ± 1.98	5.80 ± 0.95	6.10 ± 1.35	5.66 ± 0.87	5.54 ± 0.70	n.d.	9.76 ± 0.84
Probiotics	10.01 ± 0.46**	9.39 ± 0.67***	8.98 ± 1.27*	7.77 ± 1.99	7.79 ± 1.99	5.70 ± 0.88	6.07 ± 1.32	5.63 ± 0.81	5.63 ± 0.79	n.d.	9.62 ± 0.91**
Synbiotics	10.11 ± 0.42*** #	9.27 ± 0.65***	9.20 ± 1.24##	8.06 ± 2.11##	7.86 ± 2.00	5.80 ± 0.98	6.15 ± 1.40	5.69 ± 0.89	5.59 ± 0.73	n.d.	9.84 ± 0.87###

Note

Values are expressed as the mean ± standard deviation of common logarithms of the number of bacteria per 1 g feces.

lac: *B. animalis* subsp. *lactis*; lon: *B. longum* subsp. *longum*; ado: *B. adolescentis* group; cat: *B. catenulatum* or *B. pseudocatenulatum*; bre: *B. breve*; bif: *B. bifidum*; inf: *B. longum* subsp. *infantis*; den: *B. dentium*; ang: *B. angulatum*.

*n*.d.: not detected. The detection limit of quantitative PCR was 2.0 × 10^5^ cells per g of feces.

Comparisons were conducted using a paired Student's *t* test with Bonferroni's correction after logarithmic transformation of the number of each bacterial species.

*** *p* < 0.001,

** *p* < 0.01,

* *p* < 0.05 (vs. placebo);

^###^ *p* < 0.001,

^##^ *p* < 0.01,

^#^ *p* < 0.05 (vs. probiotics).

Table 4 shows the number of gut bifidobacteria in the tertile of the subjects (*n* = 20) who had lower *B. lactis* counts during the probiotic intake period. There was no significant difference in any of the species including total bifidobacteria, except for *B. lactis*, in the probiotic intake period compared with the placebo intake period. On the other hand, in the synbiotic intake period, the number of total bifidobacteria was significantly increased compared with that of the placebo and probiotic intake periods, respectively, and the number of endogenous bifidobacteria tended to increase compared with the probiotic intake period (*p* = 0.067).

**Table 4:** Number of intestinal bifidobacteria in the tertile of subjects with a smaller number of *B. lactis* during the probiotic intake period

	Total bifidobacteria	lac	lon	ado	cat	bre	bif	inf	den	ang	Endogenous bifidobacteria
Placebo	9.45 ± 1.24	5.69 ± 0.95	8.68 ± 1.84	6.58 ± 2.06	7.24 ± 2.17	5.82 ± 1.15	5.96 ± 1.35	5.52 ± 0.67	5.63 ± 0.83	*n*.d.	9.41 ± 1.33
Probiotics	9.76 ± 0.57	8.70 ± 0.64***	8.50 ± 1.78	6.50 ± 1.93	7.28 ± 2.18	5.76 ± 1.08	5.93 ± 1.29	5.52 ± 0.59	5.48 ± 0.60	*n*.d.	9.25 ± 1.38
Synbiotics	9.97 ± 0.55* #	9.08 ± 0.60***	8.88 ± 1.75#	6.56 ± 1.97	7.33 ± 2.19	5.83 ± 1.16	5.97 ± 1.38	5.54 ± 0.69	5.60 ± 0.81	*n*.d.	9.48 ± 1.36

Note

Values are expressed as the mean ± standard deviation of common logarithms of the number of bacteria per 1 g feces.

lac: *B. animalis* subsp. *lactis*; lon: *B. longum* subsp. *longum*; ado: *B. adolescentis* group; cat: *B. catenulatum* or *B. pseudocatenulatum*; bre: *B. breve*; bif: *B. bifidum*; inf: *B. longum* subsp. *infantis*; den: *B. dentium*; ang: *B. angulatum*.

n.d.: not detected. The detection limit of quantitative PCR was 2.0 × 10^5^ cells per g of feces.

Comparisons were conducted using a paired Student's *t* test with Bonferroni's correction after logarithmic transformation of the number of each bacterial species.

*** *p* < 0.001,

* *p* < 0.05 (vs. placebo);

^#^ *p* < 0.05 (vs. probiotics).

### 3.3 Frequency of defecation

The number and days of defecation are shown in Table 5. Both parameters in all periods were significantly increased compared with those in the baseline period (Table 2). However, there was no significant difference in either parameter among any of the periods.

**Table 5:** Defecation states

	Number of defecations (/week)	Days of defecation (/week)
Placebo	4.56 ± 1.23	4.23 ± 0.97
Probiotics	4.56 ± 1.23	4.27 ± 1.04
Synbiotics	4.68 ± 1.22	4.37 ± 1.04

Note

Values are expressed as the mean ± standard deviation.

## 4 DISCUSSION

In this study, we demonstrated that a synbiotic drink containing *B. lactis* GCL2505 and inulin significantly increased the total number of intestinal bifidobacteria compared with a probiotic drink containing *B. lactis* GCL2505 alone or a placebo in healthy adults with mild constipation after 2 weeks of ingestion. In the synbiotic intake period, compared to the probiotic intake period, significant increases were observed in the number of endogenous bifidobacteria. Moreover, in the tertile of the subjects (*n* = 20) who had lower *B. lactis* counts during the probiotic intake period, the number of total bifidobacteria increased significantly in the synbiotic intake period compared to the placebo and probiotic intake periods, while that in the probiotic intake period was not significantly different from the placebo intake period.

*Bifidobacterium animalis* subsp.* lactis* is considered to be a subspecies of nonendogenous bifidobacteria in the human gut (Kato et al., 2017; Turroni et al., 2009). In this study, the number of *B. lactis* was above the detection limit in the feces of only five subjects during the observation period and their levels were very low. Therefore, it was thought that most of the *B. lactis* detected during the test drink intake periods was *B. lactis* GCL2505. In this study, the ingestion of a probiotic drink containing *B. lactis* GCL2505 alone led to a significant increase in the number of total bifidobacteria in feces compared to the placebo intake period. Previously, we revealed that *B. lactis* GCL2505 reaches the intestine alive and increases the number of bifidobacteria in the intestinal tract (Ishizuka et al., 2012). In the present study, we reconfirmed the effect of *B. lactis* GCL2505 ingestion on the number of intestinal bifidobacteria.

Synbiotics, a synergistic combination of probiotics and prebiotics, are expected to have a greater effect on the health of the host than when each is used alone, and many clinical studies of synbiotics have been conducted (Asemi, Khorrami‐Rad, Alizadeh, Shakeri, & Esmaillzadeh, 2014; Childs et al., 2014; Stenman et al., 2016; Waitzberg et al., 2013). However, these reports have not shown that synbiotics have a clearly stronger effect on the numbers of intestinal bifidobacteria compared with the use of probiotics or prebiotics alone. The present study showed a greater increase in the number of total bifidobacteria by ingesting a synbiotic drink combining *B. lactis* GCL2505 and inulin than ingesting *B. lactis* GCL2505 alone or placebo. In terms of each bacterial species, the counts of *B. longum*, *B. adolescentis*, and total endogenous bifidobacteria showed a significant increase when compared with the probiotic food intake period, while no difference was observed in the number of *B. lactis*. It was reported that inulin was assimilated by some species or strains of bifidobacteria including *B. longum* and *B. adolescentis* (Roberfroid, Van Loo, & Gibson, 1998; Rossi et al., 2005). Furthermore, it was also reported that the ingestion of inulin increased the number of *B. longum* and *B. adolescentis* in the intestinal tract (Ramirez‐Farias et al., 2009). The inulin contained in the synbiotic drink in this study was thought to contribute to the increase in endogenous bifidobacteria, including *B. longum* and *B. adolescentis*. On the other hand, it was reported that *B. lactis* has relatively lower assimilability of inulin than other bifidobacteria species (Roberfroid et al., 1998). Therefore, inulin might be assimilated by some species or strains of endogenous bifidobacteria rapidly and preferentially, but did not contribute to the growth of *B. lactis* GCL2505 directly. It also means that inulin might not interfere with the proliferation of *B. lactis* GCL2505 in the gut. From these facts, it is suggested that *B. lactis* GCL2505 and inulin have little influence on each other's ability to increase the number of intestinal bifidobacteria when they are ingested concomitantly.

The amount of probiotics that arrive in the gut and their health effects often differ from one individual to another because of the individual differences in the intestinal microbiota (Mackie, Sghir, & Gaskins, 1999). This has been shown through the difference in the proliferative rates of *B. lactis* GCL2505 in the intestinal tracts of different subjects (Ishizuka et al., 2012). We hypothesized that ingestion of synbiotics containing *B. lactis* GCL2505 and inulin would increase the number of intestinal bifidobacteria, even for those subjects with low effects of probiotics. In the present study, a stratified analysis of the subjects who had low numbers of *B. lactis* subspecies during the probiotic intake period showed a significant increase in the total number of bifidobacteria in the synbiotic intake period compared with the placebo and probiotic intake periods. However, there was no significant difference in the number of total bifidobacteria between the probiotic and placebo intake periods. These results suggest that the synbiotics containing both *B. lactis* GCL2505 and inulin can increase the number of intestinal bifidobacteria even in the subjects who demonstrated modest effects from the intake of *B. lactis* GCL2505 alone. Therefore, this synbiotic approach may benefit a higher number of people compared with the usual probiotic approach, considering the variations in human intestinal microbiota.

An improvement of the intestinal microbiota resulting from the intake of probiotics or prebiotics, especially an increase in the number of intestinal bifidobacteria, often leads to a higher frequency of defecation (Matsumoto et al., 2010; Yamano et al., 2006). The greater presence of bifidobacteria in the intestinal tract promotes the production of short‐chain fatty acids, such as acetate (Aoki et al., 2017; Igarashi et al., 2017), which stimulate colonic motility (Fukumoto et al., 2003; Ono, Karaki, & Kuwahara, 2004). Some strains of *B. lactis*, including *B. lactis* strain GCL2505, were also reported to improve defecation frequency (Flach et al., 2018; Ishizuka et al., 2012; Tanaka et al., 2015; Yang et al., 2008). However, in the present study, no differences were found in a comparison between placebo and probiotic/synbiotic intake periods. Compared with baseline (Table 2), defecation frequency was significantly increased in all ingestion periods including the placebo intake period (all *p* < 0.001). Since the degree of change in the number of defecations between the baseline and each intake period at 2 weeks after ingestion was about the same level as that after the intake of *B. lactis* in previous studies (Eskesen et al., 2015; Ishizuka et al., 2012; Matsumoto et al., 2001), it was likely that *B. lactis* GCL2505 or the combination of *B. lactis* GCL2505 and inulin could reasonably exert their improvement effects on defecation frequency. On the other hand, high defecation frequency after intake of placebo was sometimes observed in past clinical studies using *B. lactis* (Eskesen et al., 2015; Nishida et al., 2004). For that reason, we determined that there was a high placebo effect for bowel movements in this study. However, the main outcome measure in the present study was the number of intestinal bifidobacteria. Bifidobacteria is one of the most beneficial bacteria for host health, and maintaining a high number of intestinal bifidobacteria is considered to be important (Rivière et al., 2016). Because the combination of *B. lactis* GCL2505 and inulin exerted a synbiotic effect on the number of intestinal bifidobacteria in this study, it is likely that synbiotics have stronger health effects than *B. lactis* GCL2505 alone.

In conclusion, the present results indicate that the ingestion of a synbiotic drink containing *B. lactis* GCL2505 and inulin increased the amount of total bifidobacteria in the human intestine compared with the ingestion of a placebo drink or a probiotic drink containing *B. lactis* GCL2505 alone. Furthermore, this synbiotic drink increased the number of total bifidobacteria in subjects who had a relatively small increase in *B. lactis* GCL2505 after the ingestion of *B. lactis* GCL2505 alone. The present findings suggest that the synbiotics containing *B. lactis* GCL2505 and inulin could be useful for the improvement of the intestinal environment of more poeple by increasing the number of bifidobacteria in the gut, which may contribute to health benefits.

## CONFLICT OF INTEREST

D. Anzawa, T. Mawatari, Y. Tanaka, M. Yamamoto, T. Genda, S. Takahashi, T. Nishijima, H. Kamasaka, and T. Kuriki are employees of Ezaki Glico Co., Ltd., which produces products using *B. lactis* GCL2505. S. Suzuki declares no conflict of interest.

## ETHICAL REVIEW

The study protocol was approved by the institutional review board of the Ethics Committee of Nihonbashi Cardiology Clinic (Tokyo, Japan).

## INFORMED CONSENT

Written informed consent was obtained from all study participants.
